# ST6GAL1 Glycoengineering Rewires Cytokine Signaling and Preserves Metabolic Fitness in CAR-T Cells Under Galectin-3-Mediated Immunosuppression

**DOI:** 10.3390/ijms27146393

**Published:** 2026-07-18

**Authors:** Lee Seng Lau, Maria Suarez, Aizada Berdalinova, Rebecca Z. Fan, Rajib Kumar Shil, Joseph Souchak, Kim Tieu, Avery D. Posey, Charles J. Dimitroff

**Affiliations:** 1 Department of Cellular and Molecular Medicine, Herbert Wertheim College of Medicine, Florida International University, Miami, FL 33199, USAjoseph.souchak@gmail.com (J.S.); 2 Department of Environmental Health Sciences, Florida International University, Miami, FL 33199, USA; zhfan@fiu.edu (R.Z.F.);; 3 Department of Systems Pharmacology and Translational Therapeutics, Perelman School of Medicine, University of Pennsylvania, Philadelphia, PA 19104, USA; 4 Corporal Michael J. Crescenz VA Medical Center, Philadelphia, PA 19104, USA

**Keywords:** CAR-T cell, ST6GAL1, Gal-3, metabolism, glycoengineering, JAK/STAT signaling, SOCS1, tumor microenvironment

## Abstract

Chimeric antigen receptor (CAR)-T cell therapy has demonstrated remarkable efficacy in hematologic malignancies; however, durable responses remain limited by tumor microenvironment (TME)-mediated immunosuppression. Galectin-3 (Gal-3), a β-galactoside-binding lectin enriched in the TME, contributes to CAR-T cell dysfunction by impairing cytotoxicity, promoting apoptosis, and altering cellular signaling. While we previously demonstrated that enforced expression of the α2,6 sialyltransferase ST6GAL1 reduces galectin binding and improves CAR-T cell function, the mechanistic basis underlying this protection remains unclear. Here, we report that Gal-3 induced a hypometabolic state in CAR-T cells characterized by reduced mitochondrial function, ATP production, and glucose utilization. In contrast, ST6GAL1-overexpressing CAR-T cells preserved metabolic fitness and functional resilience under Gal-3 stress. Additionally, Gal-3 rewired cytokine signaling by increasing IL-5 expression and dysregulating downstream pathways, whereas enforced ST6GAL1 expressing CAR-T cells exhibited increased SOCS1 and SOCS3 expression and attenuated STAT5 activation. Transcriptomic analysis of CAR-T cells from diffuse large B-cell lymphoma patients further revealed enrichment of STAT5-associated signaling and SOCS1 expression in complete responders compared to partial responders. Collectively, these findings identify glycoengineering as a promising strategy to enhance CAR-T cell persistence and function under Gal-3-mediated immunosuppressive stress.

## 1. Introduction

Chimeric antigen receptor (CAR)-T cell therapy has transformed the treatment landscape for hematological malignancies, particularly leukemias and lymphomas. CAR-T cells are genetically engineered to express a synthetic receptor composed of an extracellular single-chain variable fragment (scFv) derived from antibody variable regions linked to transmembrane and intracellular signaling domains. This design enables the major histocompatibility complex (MHC)-independent recognition of tumor-associated antigens and redirects T cell cytotoxicity toward malignant cells [1].

The remarkable clinical success of CAR-T therapy has led to the U.S. Food and Drug Administration (FDA) approval of multiple anti-CD19 CAR-T cell products for the treatment of B-cell malignancies [2]. However, increasing evidence indicates that the tumor microenvironment (TME) plays a central role in limiting CAR-T cell persistence and function [3].

Several mechanisms contribute to CAR-T cell failure, including tumor heterogeneity and plasticity leading to antigen loss [4], expansion and persistence [5,6], chronic antigen-exposure-driven T cell dysfunction [7,8], and the immunosuppressive TME [9,10]. Among these, immunosuppressive factors within the TME are critical regulators of T cell dysfunction. In particular, galectins, a family of β-galactoside-binding lectins, play a central role in tumor immune evasion. Galectins can bind glycan structures on T cells and modulate immune responses by activating co-inhibitory receptors, disrupting costimulatory signaling, regulating T cell activation and cytokine secretion, skewing differentiation, and impairing immune cell survival [11].

Among these, galectin-3 (Gal-3) has emerged as a key regulator of T cell activity. Gal-3 is frequently elevated in hematologic malignancies and contributes to immune evasion by binding cell surface glycans, leading to impaired cytotoxicity, altered cytokine production, and increased apoptosis [12,13].

We have previously demonstrated that CAR-T cells exhibit increased susceptibility to galectin binding due to altered glycosylation, including reduced expression of the α2,6 sialyltransferase ST6GAL1 [14]. Aberrant glycosylation is increasingly recognized as a key regulator of T cell function and immune checkpoint signaling [15,16]. Enforced expression of ST6GAL1 reduced Gal-3-binding and improved CAR-T cell survival and function. However, the mechanisms by which glycoengineering confers resistance to TME-mediated suppression remain poorly defined.

In our previous study, we also identified IL-5 as a Gal-3-responsive cytokine that was significantly induced following Gal-3 exposure and attenuated by ST6GAL1 glycoengineering [14]. Because IL-5 signals through the JAK/STAT pathway, these findings suggested that dysregulated cytokine signaling may contribute to Gal-3-mediated CAR-T cell dysfunction. However, the intracellular signaling mechanisms linking Gal-3 exposure to altered cytokine responses and CAR-T cell metabolic fitness remain poorly understood.

Emerging evidence indicates that metabolic fitness and cytokine signaling are fundamental determinants of CAR-T cell persistence, expansion, and antitumor efficacy [17]. Mitochondrial dysfunction and impaired bioenergetic capacity have been associated with T cell exhaustion and therapeutic failure, whereas balanced cytokine signaling is essential for maintaining effector function and long-term persistence [18,19]. However, whether Gal-3 influences these pathways in CAR-T cells and whether ST6GAL1-mediated glycoengineering can preserve metabolic and signaling homeostasis remain unknown.

In this study, we investigated the signaling mechanisms underlying ST6GAL1-mediated protection against Gal-3-induced immunosuppression by examining alterations in cytokine signaling, STAT5 activation, SOCS1 expression, and CAR-T cell metabolic fitness under Gal-3-mediated stress. Metabolic fitness is a critical determinant of CAR-T cell persistence and antitumor activity [20,21,22]. We further evaluated the clinical relevance of these findings using transcriptomic datasets from CAR-T-treated patients. Our results demonstrated that glycoengineering not only protects CAR-T cells from extracellular Gal-3 suppression but also rewires intracellular signaling and metabolic pathways to promote functional resilience.

## 2. Results

### 2.1. ST6GAL1 Altered Cytokine Signaling Gene Expression in CAR-T Cells Following Gal-3 Exposure

**Figure 1:**
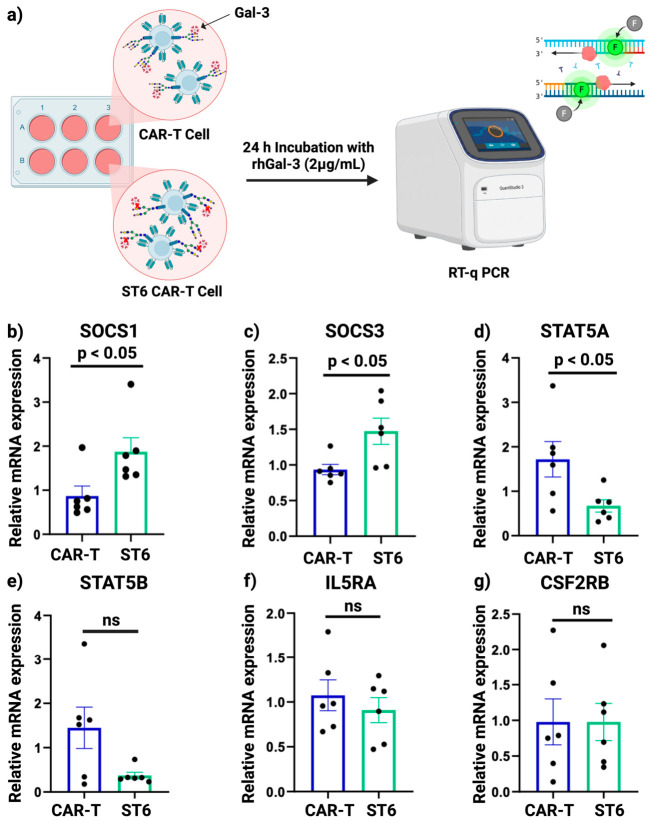
ST6GAL1 altered cytokine signaling gene expression in CAR-T cells following Galectin-3 exposure. (**a**) CAR-T and ST6 CAR-T cells were cultured in the presence or absence of rhGal-3 (2 µg/mL) for 24 h. Following incubation, cells were harvested and gene expression was assessed via RT-qPCR. (**b**–**g**) Relative mRNA expression of cytokine signaling genes in CAR-T and ST6 CAR-T cells following rhGal-3 exposure. ST6GAL1 overexpression significantly increased SOCS1 and SOCS3 mRNA levels and decreased STAT5A mRNA levels compared to control CAR-T cells. No significant differences were observed for STAT5B, IL5RA, or CSF2RB. Data were presented from n = 6 independent donors and graphed as the mean ± SEM (*p* < 0.05). Statistical significance was determined using a Mann–Whitney test (Created in BioRender. Suarez, M. (2026) https://BioRender.com/1i3k1l5 (accessed on 17 June 2026)).

To investigate whether ST6GAL1-mediated glycoengineering alters cytokine signaling pathways associated with Gal-3-induced CAR-T cell dysfunction, anti-CD19 CAR-T cells and enforced ST6GAL1-expressing CAR-T cells (ST6 CAR-T cells), confirmed via flow cytometry for increased SNA-α2,6 sialylation binding (Supplementary Figure S1a), were incubated with recombinant human (rh) Gal-3 at a 2 µg/mL for 24 h prior to the analysis of JAK/STAT pathway-associated genes via RT-qPCR (Figure 1a). ST6 CAR-T cells exhibited significantly increased expression of the suppressor of cytokine signaling molecules SOCS1 and SOCS3 compared with conventional CAR-T cells following rhGal-3 exposure (Figure 1b,c) (*p* < 0.05). In contrast, *STAT5A* expression was significantly reduced in ST6 CAR-T cells relative to CAR-T cell controls (Figure 1d) (*p* < 0.05), whereas *STAT5B* expression showed a downward trend but did not reach statistical significance (Figure 1e). Expression of the IL-5 receptor α-chain (IL5RA) and the common β-chain receptor (CSF2RB) was not significantly altered between groups (Figure 1f,g). These findings demonstrated that ST6GAL1 overexpression promotes a transcriptional profile characterized by enhanced SOCS expression and reduced STAT5 signaling following Gal-3 stimulation.

### 2.2. ST6GAL1 Attenuated Gal-3-Induced STAT5 Activation and Promoted SOCS1 Feedback Signaling in CAR-T Cells

**Figure 2:**
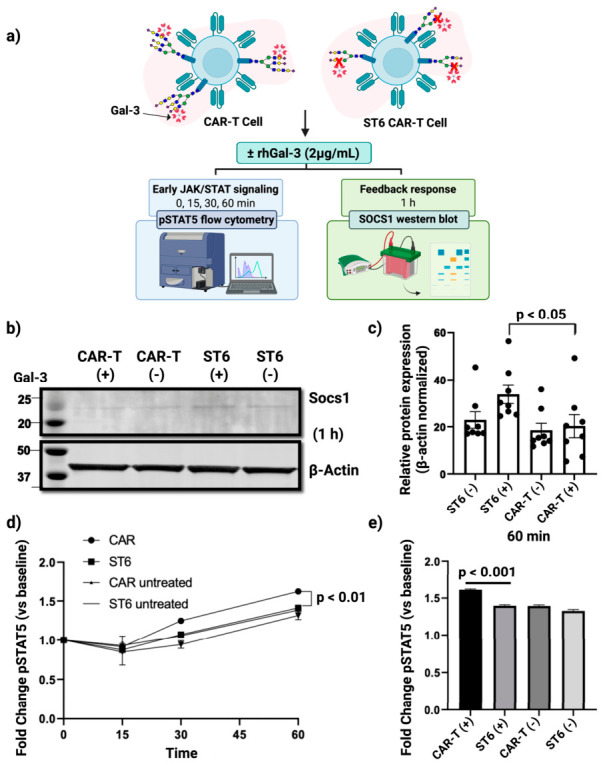
ST6GAL1 attenuated Gal-3-induced STAT5 activation and promoted SOCS1 feedback signaling in CAR-T cells (**a**) CAR-T and ST6 CAR-T cells were treated with and without rhGal-3 (2 µg/mL). Early JAK/STAT5 signaling was assessed by measuring phosphorylated STAT5 via flow cytometry at 0, 15, 30, and 60 min post-treatment. The feedback response was evaluated at 1 h by measuring SOCS1 protein levels via Western blotting. (**b**) Representative Western blot showing SOCS1 protein expression in ST6 CAR-T and CAR-T cells treated with or without Gal-3 for 1 h. β-Actin was used as a loading control. (**c**) Quantification of SOCS1 protein expression normalized to β-Actin across all conditions. ST6 CAR-T cells treated with Gal-3 showed significantly elevated SOCS1 protein compared to Gal-3-treated CAR-T cells, presented from n = 8 independent donors and graphed as the mean ± SEM (*p* < 0.05). Statistical significance was determined using a Wilcoxon test. (**d**) Time-course analysis of pSTAT5 fold change relative to the baseline (0 min) in CAR-T and ST6 CAR-T cells treated with or without rhGal-3, measured at 0, 15, 30, and 60 min via flow cytometry. CAR-T cells treated with rhGal-3 exhibited a significantly greater increase in pSTAT5 over time compared to ST6 CAR-T cells, presented from n = 4 independent donors and graphed as the mean ± SEM (*p* < 0.01). Statistical significance was determined using a two-way ANOVA with Sidak’s multiple comparisons test. (**e**) Fold change in pSTAT5 at the 60 min endpoint across all four conditions. CAR-T cells treated with rhGal-3 showed significantly higher pSTAT5 levels compared to ST6 CAR-T cells treated with rhGal-3, indicating that ST6GAL1 attenuated Gal-3-induced STAT5 phosphorylation, presented from n = 4 independent donors and graphed as the mean ± SEM (*p* < 0.001). Statistical significance was determined using a two-way ANOVA (Created in BioRender. Suarez, M. (2026) https://BioRender.com/0kx5q6v (accessed on 17 June 2026)).

To determine whether the transcriptional changes observed in ST6 CAR-T cells translated to altered STAT5 signaling, CAR-T and ST6 CAR-T cells were exposed to rhGal-3 for 1 h at 2 µg/mL and evaluated for SOCS1 protein expression and STAT5 phosphorylation (Figure 2a). Western blot analysis performed after 1 h of rhGal-3 stimulation demonstrated significantly increased SOCS1 protein expression in ST6 CAR-T cells compared with conventional CAR-T cells (Figure 2b,c) (*p* < 0.05). Because SOCS proteins function as negative regulators of cytokine signaling, we next evaluated STAT5 phosphorylation kinetics via phospho-flow cytometry. Gal-3 induced a time-dependent increase in pSTAT5 levels in both CAR-T and ST6 CAR-T cells; however, conventional CAR-T cells exhibited significantly greater STAT5 activation at later time points (Figure 2d). At 60 min, rhGal-3-treated CAR-T cells displayed significantly higher pSTAT5 levels compared with ST6 CAR-T cells (Figure 2e) (*p* < 0.001). Collectively, these findings indicated that ST6GAL1 expression is associated with attenuated Gal-3-mediated STAT5 activation and increased SOCS1 expression, consistent with enhanced SOCS-dependent feedback regulation.

### 2.3. ST6GAL1 Glycoengineering Preserved CAR-T Cell Metabolic Fitness in Gal-3-Rich Environments

**Figure 3:**
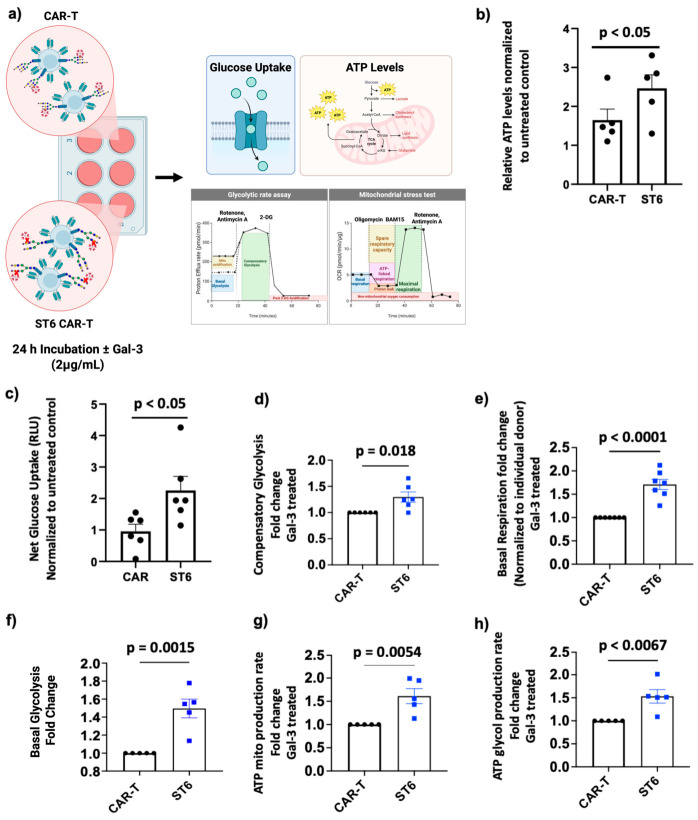
ST6GAL1 glycoengineering preserved CAR-T cell metabolic fitness in Gal-3-rich environments. (**a**) CAR-T and ST6 CAR-T cells were incubated with Gal-3 (2 µg/mL) for 24 h and metabolically profiled using Seahorse Analyzers, including the mitochondrial stress test, glycolytic rate assay, glucose uptake, and ATP quantification assays. (**b**,**c**) ST6 CAR-T cells maintained significantly higher relative ATP levels and net glucose uptake compared to CAR-T cells following Gal-3 treatment, each normalized to untreated controls (*p* < 0.05). (**d**–**f**) Under Gal-3 treatment, ST6 CAR-T cells showed significantly greater fold changes in compensatory glycolysis, basal respiration, and basal glycolysis relative to CAR-T cells (*p* = 0.018, *p* < 0.0001, and *p* = 0.0015, respectively). (**g**,**h**) ST6 CAR-T cells preserved significantly higher mitochondrial ATP production and glycolytic ATP production rates compared to CAR-T cells in Gal-3-treated conditions (*p* = 0.0054 and *p* = 0.0067, respectively). Data are presented from n = 6 independent donors as the mean ± SEM. Statistical significance was determined using an unpaired *t*-test. (Created in BioRender. Suarez, M. (2026) https://BioRender.com/2qm25gl (accessed on 17 June 2026)).

Given the established relationship between cytokine signaling and cellular metabolism, we next investigated whether ST6GAL1 overexpression influences metabolic fitness following Gal-3 exposure. CAR-T and ST6 CAR-T cells were incubated with rhGal-3 for 24 h using the same concentration of 2 µg/mL prior to analysis of ATP production, glucose uptake, and bioenergetic function using Seahorse extracellular flux assays (Figure 3a).

ST6 CAR-T cells maintained significantly higher intracellular ATP levels compared with conventional CAR-T cells following Gal-3 treatment (Figure 3b) (*p* < 0.05). Likewise, glucose uptake was significantly increased in ST6 CAR-T cells relative to CAR-T cell controls (Figure 3c) (*p* < 0.05).

Futhermore, independent Seahorse analysis demonstrated that under basal conditions, ST6GAL1 overexpression did not significantly alter metabolic activity compared with conventional CAR-T cells (Supplementary Figure S1b). However, treatment with rhGal-3 induced substantial metabolic suppression, characterized by significant reductions in basal mitochondrial respiration (Supplementary Figure S1c) (*p* = 0.0074), basal glycolysis (Supplementary Figure S1d) (*p* = 0.0075), and overall ATP production rates (Supplementary Figure S1e) (*p* = 0.0024). Importantly, neither maximal respiration, absolute spare respiratory capacity, nor coupling efficiency were diminished, indicating that the Gal-3-induced metabolic reduction is distinct from overt mitochondrial dysfunction.

While substantial inter-donor variability was observed—particularly within the Gal-3-treated cohorts—a paired analysis controlling for donor origin revealed the protective role of ST6. When compared to matched, rhGal-3-treated controls from the same donor, ST6 CAR-T cells exhibited significantly improved compensatory glycolysis (Figure 3d) (*p* = 0.0118), basal respiration (Figure 3e) (*p* < 0.0001), and (Figure 3f) basal glycolysis (*p* = 0.0015). This rescue was accompanied by increased ATP production from both mitochondrial (Figure 3g) (*p* = 0.0054) and glycolytic (Figure 3h) (*p* = 0.0067) sources, demonstrating that ST6 confers robust metabolic protection against Gal-3. Collectively, these findings demonstrated that ST6GAL1-mediated glycoengineering protects CAR-T cells from Gal-3-induced metabolic suppression and preserves bioenergetic fitness under immunosuppressive conditions.

### 2.4. Gal-3 Impaired CAR-T Cell Proliferation, and JAK Inhibition Partially Restored Cytotoxic Function Under Gal-3-Mediated Stress

**Figure 4:**
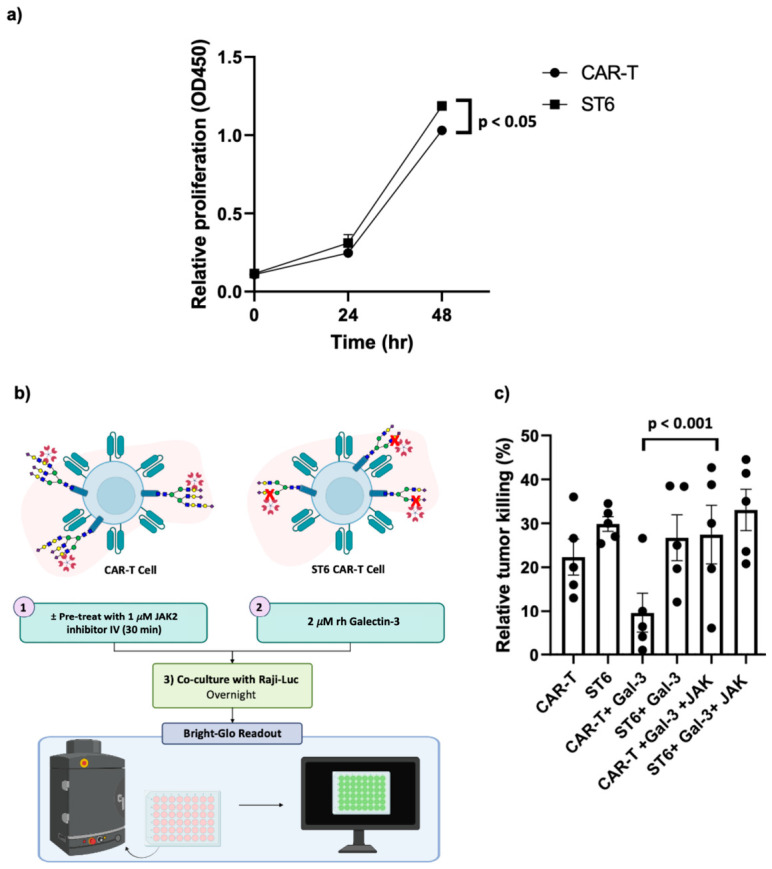
Gal-3 impaired CAR-T cell proliferation, and JAK inhibition partially restored cytotoxic function under Gal-3-mediated stress. (**a**) Relative proliferation of CAR-T and ST6 CAR-T cells measured based on the absorbance at 0, 24, and 48 h in the presence of rhGal-3. ST6 CAR-T cells demonstrated significantly greater proliferative capacity at 48 h compared to CAR-T cells, presented from n = 4 independent donors as the mean ± SEM (*p* < 0.05). Statistical significance was determined using mixed-effect analysis. (**b**) Schematic of the cytotoxicity assay. CAR-T and ST6 CAR-T cells were pre-treated with or without 1 µM JAK inhibitor for 30 min, followed by exposure to 2 µg/mL rhGal-3 and overnight co-culture with Raji-Luc target cells. Cytotoxicity was assessed based on the Bright-Glo luminescence readout. (**c**) Relative tumor killing (%) across six conditions, untreated CAR-T and ST6 CAR-T cells, rhGal-3–treated CAR-T and ST6 CAR-T cells, and rhGal-3–treated CAR-T and ST6 CAR-T cells with the JAK inhibitor (JAK). Gal-3 treatment markedly reduced CAR-T cytotoxicity, while JAK inhibition significantly rescued tumor killing in Gal-3-treated CAR-T cells, presented from n = 5 independent donors as the mean ± SEM (*p* < 0.001). Statistical significance was determined using a repeated-measures one-way ANOVA with Sidak’s multiple comparisons test (created in BioRender. Suarez, M. (2026) https://BioRender.com/t28uxro (accessed on 17 June 2026)).

To evaluate the functional consequences of the ST6GAL1-mediated modulation of STAT5 signaling, CAR-T cell proliferation and cytotoxic activity were assessed following rhGal-3 exposure (Figure 4a). Consistent with the metabolic advantages observed in ST6 CAR-T cells, enforced ST6GAL1 expression significantly enhanced CAR-T cell proliferation compared with conventional CAR-T cells in the presence of Gal-3 (Figure 4a) (*p* < 0.05).

To further investigate whether JAK/STAT signaling contributes to Gal-3-induced CAR-T cell dysfunction, the effects of pharmacologic JAK inhibition on CAR-T cell cytotoxicity were examined. Consistent with our previous findings, Gal-3 markedly reduced the tumor-killing capacity of conventional CAR-T cells. Importantly, treatment with a JAK inhibitor (Figure 4b) partially restored CAR-T cell cytotoxicity in the presence of Gal-3, resulting in levels comparable to those observed in ST6 CAR-T cells (Figure 4c) (*p* < 0.001). JAK inhibition also modestly enhanced the cytotoxic activity of ST6 CAR-T cells. These findings support a role for aberrant JAK/STAT signaling in Gal-3-induced CAR-T cell dysfunction and are consistent with the possibility that the attenuation of this pathway contributes to the protective effects of ST6GAL1 overexpression.

### 2.5. Independent CAR-T Cell Transcriptomic Profiling Supported Signaling and Metabolic Rewiring Associated with Clinical Outcomes

**Figure 5:**
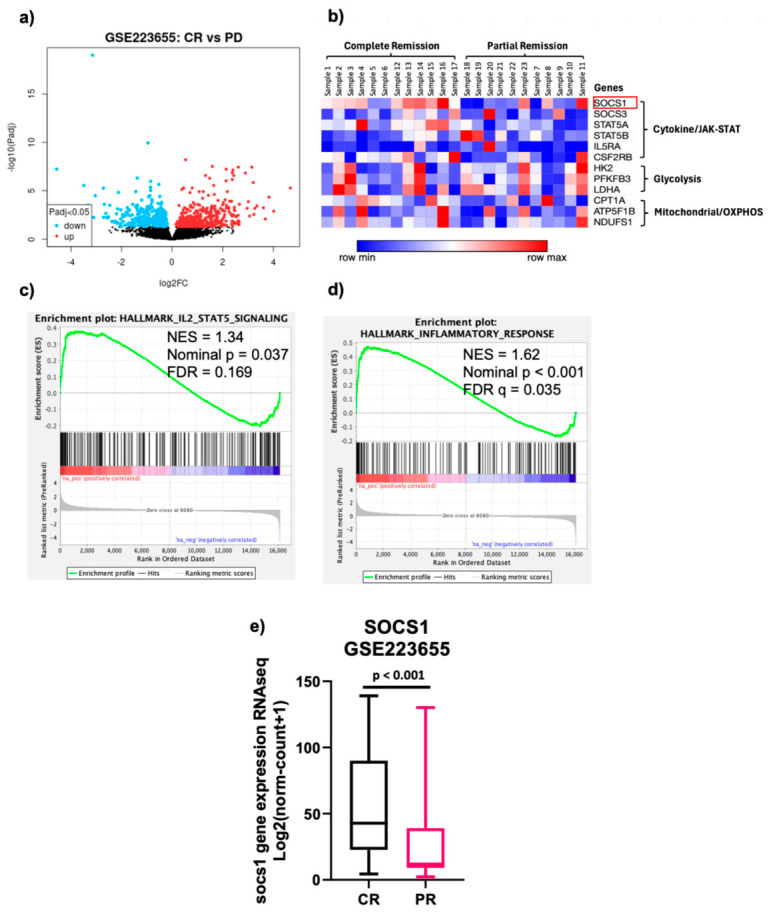
Independent CAR-T cell transcriptomic profiling supported signaling and metabolic rewiring associated with clinical outcomes. (**a**) Volcano plot of differentially expressed genes from the GSE223655 dataset comparing patients achieving complete remission (CR) versus patients with progressive disease (PD) following CD19/CD20 TanCAR-T (Tandem Chimeric Antigen Receptor T cell) therapy for DLBCL. Significantly upregulated genes are shown in red, and downregulated genes are shown in blue (adjusted *p* < 0.05). (**b**) Heatmap showing relative expression of selected genes involved in cytokine/JAK-STAT signaling (SOCS1, SOCS3, STAT5A, STAT5B, IL5RA, CSF2RB), glycolysis (HK2, PFKFB3, LDHA, CPT1A), and mitochondrial oxidative phosphorylation (ATP5F1B, NDUFS1) across n = 12 complete remission (CR) and n = 11 partial remission (PR) patient samples. Gene expression values were row normalized and displayed as relative expression (blue, low; red, high). (**c**) Gene set enrichment analysis (GSEA) demonstrating enrichment of the HALLMARK_IL-2_STAT5_SIGNALING pathway in CR compared with PD samples. (**d**) GSEA demonstrating enrichment of the HALLMARK_INFLAMMATORY_RESPONSE pathway in CR compared with PD samples. (**e**) Box-and-whisker plot showing significantly increased SOCS1 expression in patients achieving CR compared with PR patients. Boxes represent the interquartile range, center lines indicate median values, and whiskers represent minimum-to-maximum values. Statistical significance was determined from normalized RNA sequencing counts (*p* < 0.001). GSE223655 data were obtained from a published cohort of patients with relapsed/refractory DLBCL treated with CD19/CD20 TanCAR-T cells. Gene set enrichment analysis was performed using preranked differential expression data. CR, complete remission; PR, partial remission; PD, progressive disease; GSEA, gene set enrichment analysis.

To assess the clinical relevance of our findings, we analyzed the publicly available CAR-T transcriptomic dataset GSE223655 comparing patients achieving complete remission (CR) with those exhibiting progressive disease (PD) following CAR-T therapy. Differential expression analysis identified substantial transcriptional differences between clinical responders and non-responders (Figure 5a).

Heatmap analysis revealed the differential expression of multiple genes associated with cytokine signaling, glycolysis, and mitochondrial metabolism, including *SOCS1*, *SOCS3*, *STAT5A*, *STAT5B*, *IL5RA*, *CSF2RB*, *HK2*, *PFKFB3*, *LDHA*, *CPT1A*, *ATP5F1B*, and *NDUFS1* (Figure 5b). To ensure data quality and comparability across samples, normalized gene expression distributions from the GSE223655 dataset were examined. Boxplot analysis demostrated a highly similar expression distribution across CR and PD samples following normalization, indicating the successful correction of technical variation and suitability for downstream differential expression and gene set enrichment analyses (Supplementary Figure S1f). Gene set enrichment analysis (GSEA) demonstrated significant enrichment of the HALLMARK_IL2_STAT5_SIGNALING pathway in complete responders (Figure 5c) (NES = 1.34, FDR = 0.169). A trend toward reduced oxidative phosphorylation was observed in PD samples; however, enrichment did not reach statistical significance (Supplementary Figure S1g) (NES = −1, 23, nominal *p* = 0.066, FDR q = 0.260). Similarly, the HALLMARK_INFLAMMATORY_RESPONSE pathway was significantly enriched in complete responders (Figure 5d) (NES = 1.62, FDR = 0.035). Consistent with our experimental observations, SOCS1 expression was significantly elevated in complete responders compared with patients with progressive disease (Figure 5e) (*p* < 0.001).

Together, these findings support the clinical relevance of STAT5/SOCS signaling programs in determining CAR-T therapeutic responses and are consistent with a model whereby ST6GAL1-mediated glycoengineering promotes CAR-T cell persistence and function through its glyco-protection by Gal-3-dependent cytokine signaling networks.

## 3. Discussion

CAR-T cell therapy has revolutionized the treatment of hematologic malignancies; however, durable clinical responses remain limited by inadequate persistence and the immunosuppressive effects of the tumor microenvironment (TME) [1]. Increasing evidence indicates that galectins represent a major class of soluble immunoregulatory molecules capable of impairing T cell activation, survival, and effector function [11,23,24].

These findings build upon our previous observations that CAR-T cells possess reduced ST6GAL1 expression and diminished α2,6-sialylation, resulting in enhanced Gal-3 binding, increased apoptosis, and impaired antitumor function. In that study, enforced ST6GAL1 expression reduced Gal-3 binding and improved CAR-T cell persistence and efficacy in vivo. The current work extends these findings by identifying alterations in STAT5/SOCS signaling and metabolic fitness as potential downstream pathways associated with the protective effects of glycoengineering through which glycoengineering protects CAR-T cells from Gal-3-mediated dysfunction.

One of the most notable findings of this study is the identification of altered STAT5/SOCS signaling following Gal-3 exposure on CAR-T cells. Enforcing ST6GAL1 expression in CAR-T cells exhibited increased expression of SOCS1 and SOCS3 together with reduced STAT5A expression and a trend toward lower STAT5B expression. These transcriptional findings were further supported at the protein level, where ST6 CAR-T cells displayed enhanced SOCS1 expression and reduced STAT5 phosphorylation following Gal-3 stimulation. SOCS proteins function as classical negative regulators of cytokine signaling and serve as important feedback mechanisms that limit excessive JAK/STAT pathway activation [25]. Although STAT5 signaling is essential for T cell proliferation, survival, and effector differentiation [26], persistent or dysregulated STAT5 activation has been associated with altered T cell functionality and differentiation.

Cellular metabolism is increasingly recognized as a critical determinant of CAR-T cell persistence and therapeutic efficacy [22,27]. Activated T cells rely heavily on glycolysis to support proliferation, cytokine production, and cytotoxic activity, whereas mitochondrial fitness is required for sustained antitumor responses [28,29]. Here, we demonstrate that Gal-3 exposure induced profound metabolic suppression characterized by reduced basal respiration, glycolysis, compensatory glycolysis, and ATP production. Notably, the suppressive effects of Gal-3 were most pronounced within the glycolytic compartment, suggesting that glycolysis represents a major metabolic vulnerability of CAR-T cells exposed to galectin-rich environments.

A particularly important finding of this study is that ST6GAL1-mediated glycoengineering protected CAR-T cells from Gal-3-induced metabolic suppression. By limiting Gal-3 engagement at the cell surface, ST6GAL1 expression may preserve the critical signaling pathways required for maintaining metabolic fitness, thereby supporting CAR-T cell persistence and function within hostile tumor microenvironments. These observations are consistent with emerging evidence demonstrating that transcriptional and metabolic fitness are critical determinants of durable CAR-T cell responses [22].

Importantly, an analysis of an independent clinical CAR-T transcriptomic dataset supported the translational relevance of our findings. Complete responders demonstrated the enrichment of IL-2/STAT5 signaling and inflammatory response pathways together with elevated SOCS1 expression. The enrichment of inflammatory response pathways in complete responders further suggests that the preservation of functional immune activation programs may contribute to durable CAR-T cell responses. These findings further support a role for cytokine signaling and metabolic regulation in determining CAR-T cell therapeutic outcomes.

**Figure 6:**
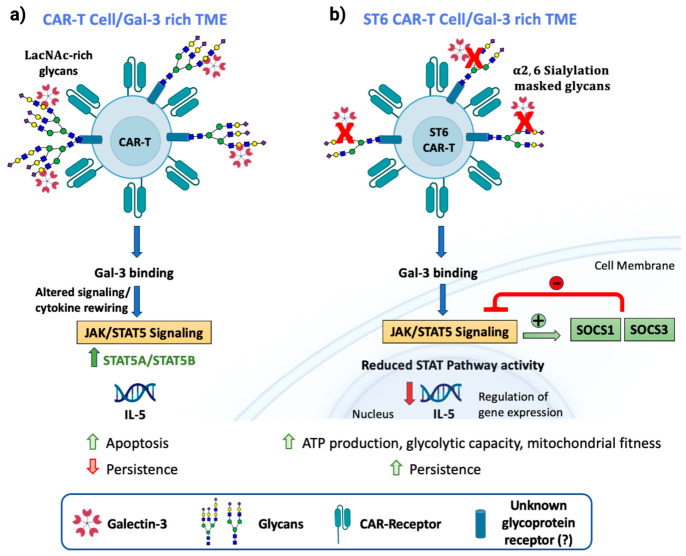
Proposed working model illustrating how ST6GAL1 glycoengineering may modulate Gal-3-mediated signaling in CAR-T cells. In conventional CAR-T cells (**a**), LacNAc-rich glycans on the cell surface permitted Gal-3-binding, leading to altered cytokine signaling and upregulated JAK/STAT5 pathway activity, including increased STAT5A/STAT5B expression and downstream IL-5 gene expression. (**b**) In ST6 CAR-T cells, α2,6-sialylation masked surface glycans, blocking Gal-3-binding. This promoted the upregulation of SOCS1 and SOCS3, which acted as negative feedback regulators to inhibit JAK/STAT signaling, reduce STAT pathway activity, and suppress IL-5 gene expression in the nucleus. This model integrated the experimental findings of the present study with previously reported STAT5/SOCS signaling mechanism and represents a proposed mechanistic framework rather than a directly demonstrated signaling cascade (created in BioRender. Suarez, M. (2026) https://BioRender.com/eijorcf (accessed on 17 June 2026)).

Collectively, these findings support a working model in which ST6GAL1-mediated α2,6-sialylation is associated with reduced Gal-3-mediated STAT5 activation, increased SOCS1 expression, and the preservation of CAR-T cell metabolic fitness, thereby promoting CAR-T cell persistence and antitumor activity. This model integrates the experimental findings of the present study with previously described STAT5–SOCS regulatory mechanisms. Although these observations support a potential mechanistic framework, the causal relationships among these signaling events remain to be experimentally established (Figure 6).

Despite these promising findings, several limitations should be considered when interpreting the present study. First, the mechanistic relationship among Gal-3 binding, STAT5 activation, SOCS1 induction, and metabolic suppression remain incompletely defined. While our data demonstrate that ST6GAL1 expression attenuates Gal-3-induced STAT5 activation, increases SOCS1 expression, and preserves metabolic fitness, these experiments do not establish direct causal relationships among these events. Instead, the proposed signaling model integrates our experimental observations with previously described STAT5–SOCS regulatory mechanisms. Future studies utilizing the genetic manipulation of STAT5 and SOCS family members will be necessary to define the mechanistic links underlying this proposed signaling pathway. Second, the current work employed recombinant Gal-3 as a model of a galectin-rich tumor microenvironment and, therefore, may not fully capture the complexity of galectin-mediated interactions occurring across the range of galectin concentrations present within the tumor microenvironment in vivo. Furthermore, the transcriptomic analysis reflects CAR-T cells from patients following successful therapy and therefore represents a distinct biological context from the acute Gal-3-mediated immunosuppressive conditions modeled in vitro. Therefore, the extent to which these transcriptional signatures are conserved across different CAR constructs, costimulatory domains, manufacturing platforms, and disease indications remains unclear. Future studies evaluating ST6GAL1-mediated glycoengineering across multiple CAR-T cell designs, including both CD28- and 4-1BB-based constructs, as well as CAR-T cells targeting solid tumors, will be important to determine the broader applicability of these findings. Finally, although ST6GAL1-mediated glycoengineering protected CAR-T cells from Gal-3-induced metabolic dysfunction, the precise mechanisms linking glycan remodeling to cellular metabolism remain unknown. Future studies will be required to determine whether altered glycan utilization and metabolic pathway regulation contribute to the enhanced persistence and antitumor activity observed in ST6GAL1-engineered CAR-T cells, as cellular metabolic fitness has emerged as a critical determinant of CAR-T cell therapeutic outcomes. Collectively, these studies will further define the translational potential of glycoengineering strategies for next-generation adoptive cellular therapies.

## 4. Materials and Methods

### 4.1. Cell Lines

The human B-lymphoma Raji cell line expressing luciferase (Raji-Luc^+^) was purchased from ATCC (Manassas, VA, USA), and cells were cultured in RPMI-1640/10% FBS. Cell cultures were regularly validated to be *Mycoplasma*-free. 

### 4.2. Plasmid Amplification and Lentiviral Production

Anti-CD19 CAR (CD19 scFv/4-1BB/CD3ζ) and *ST6GAL1*/anti-CD19 (*ST6GAL1*-P2A-CD19 scFv/4-1BB/CD3ζ) (transfer plasmids) were kindly provided by Dr. Avery D. Posey Jr. The plasmids were amplified via heat shock transformation using Stbl3 cells (Invitrogen, Waltham, MA, USA) and subsequently isolated with a Maxi Prep kit (QIAGEN, Germantown, MD, USA) according to the manufacturer’s instructions. Lentiviral vectors were produced by transfecting HEK 293T cells (ATCC, Manassas, VA, USA), which were grown to 70% confluency at 37 °C in a 5% CO_2_ incubator, with the transfer plasmids along with the LV-MAX Lentiviral mix (Gibco™, Waltham, MA, USA) and the lentiviral packaging plasmids—pRSV.REV (Rev expression vector), pMDLg/p.RRE (Gag/Pol expression plasmid, Addgene #12251, Watertown, MA, USA), and pVSV-G (VSV glycoprotein expression vector)—and these were combined with Opti-MEM (Thermo Fisher Scientific, Waltham, MA, USA) at a 15:18:18:7 mass–unit ratio. This DNA mixture was then added, at a 1:1 volume ratio, to a pre-warmed mixture of Opti-MEM and Lipofectamine 2000 (Thermo Fisher Scientific) and incubated at room temperature for 10 min before being added to the HEK 293T cells in fresh culture medium. Viral supernatants were collected at 24 and 48 h post-transfection, passed through 0.45 μm filters, and concentrated using an Amicon^®^ Ultra Centrifugal Filter (Millipore, Burlington, MA, USA). The concentrated lentivirus was stored at −80 °C, and the viral titer was determined using the Lenti-X™ Provirus Quantitation Kit (Takara Bio, San Jose, CA, USA). 

### 4.3. Isolation, Transduction, and Expansion of Primary Human T Cells

Peripheral blood mononuclear cells (PBMCs) were isolated from normal healthy donor leukopacks (OneBlood, Inc., Miami, FL, USA) using Ficoll-Paque (GE Healthcare, Marlborough, MA, USA) gradient centrifugation. PBMC aliquots were thawed, washed, and subjected to immunomagnetic cell separation using the Pan T cell Isolation Kit (Miltenyi Biotec, Gaithersburg, MD, USA). Naïve T cells were stimulated with ImmunoCult^TM^ Human CD3/CD28 T cell Activator (Stem Cell Technologies; 25 μL/mL, Vancouver, BC, Canada) and transduced one day after activation with lentiviral vectors at a multiplicity of infection (MOI) of 3. To support T cell expansion, human IL-2 (R&D Systems, Minneapolis, MN, USA) was added every other day for 10 days at a final concentration of 100 IU/mL. 

### 4.4. RT-qPCR Analysis

To investigate the effects of Gal-3 on cytokine signaling pathways in CAR-T cells, anti-CD19 CAR-T cells and ST6GAL1-overexpressing CAR-T cells (ST6 CAR-T cells) were cultured at 2 × 10^5^ cells/mL in complete medium and treated with recombinant human galectin-3 (rhGal-3; 2 μg/mL; R&D Systems) in 24-well plates for 24 h at 37 °C and 5% CO_2_. RT-qPCR analysis was conducted on RNA extracted from CAR-T and ST6 CAR-T cells. A concentration of 2 µg/mL of recombinant human galectin-3 was selected based on our previous work demonstrating the reproducible Gal-3-mediated suppression of CAR-T cell function under these experimental conditions (Lau et al., 2026 [14]). cDNA was synthesized, and real-time quantitative RT-PCR was performed to assess genes related to galectin ligand-building/suppressing ligand synthesis, *SOCS1*, *SOCS3*, *STAT5A*, *STAT5B*, *IL5RA*, and *CSF2RB*. Data were normalized to the housekeeping gene 18S, and relative transcript levels were analyzed using the 2^(−ΔΔCt)^ method from 6 independent experiments [30].

### 4.5. Immunoblotting

CAR-T cells and ST6GAL1-overexpressing CAR-T cells (ST6 CAR-T cells) were cultured in the presence of recombinant human galectin-3 (rhGal-3; 2 μg/mL) for 1 h at 37 °C. Cells were subsequently lysed in Pierce™ RIPA buffer (Thermo Fisher Scientific) supplemented with a protease and phosphatase inhibitor cocktail (Thermo Fisher Scientific). Following incubation on ice for 30 min, lysates were centrifuged at 10,000 rpm for 10 min at 4 °C, and protein concentrations were determined using the Pierce™ BCA Protein Assay Kit (Thermo Fisher Scientific) according to the manufacturer’s instructions.

Equal amounts of protein were mixed with Laemmli sample buffer (Bio-Rad, Hercules, CA, USA), denatured at 95 °C for 5 min, and separated via SDS-PAGE using 4–12% gradient gels (Bio-Rad). Proteins were transferred onto polyvinylidene fluoride (PVDF) membranes (Millipore), blocked for 1 h at room temperature using Intercept^®^ (TBS) Blocking Buffer (LI-COR Biosciences, Lincoln, NE, USA), and incubated overnight at 4 °C with primary antibodies against SOCS1 (Cell signaling, Danvers, MA, USA) (E4K7Q) and ß-actin. Membranes were washed and incubated with IRDye^®^ secondary antibodies (LI-COR Biosciences) for 1 h at room temperature. Protein bands were visualized using an Odyssey^®^ CLx Imaging System (LI-COR Biosciences), and band intensities were quantified using Image Studio software (version.5.2) (LI-COR Biosciences).

### 4.6. Intracellular Phospho-STAT5 Flow Cytometric Analysis

CAR-T cells and ST6GAL1-overexpressing CAR-T cells (ST6 CAR-T cells) were resuspended at 0.5–1 × 10^6^ cells per condition in pre-warmed complete culture medium and rested for 30–60 min at 37 °C. Cells were then stimulated with recombinant human galectin-3 (rhGal-3; 2 μg/mL) for 0, 15, 30, or 60 min at 37 °C. Following stimulation, cells were fixed via the addition of pre-warmed Fixation Buffer (BioLegend; Cat. No. 420801, San Diego, CA, USA) and incubated for 15 min at 37 °C to preserve the phosphorylation status. Cells were subsequently washed once with PBS and permeabilized in 400 μL of cold True-Phos™ Perm Buffer (BioLegend; Cat. No. NC1166187) for at least 60 min at −20 °C.

Following permeabilization, cells were washed twice with Cell Staining Buffer and stained with APC-conjugated anti-phospho-STAT5 (Tyr694) antibody (BioLegend) for 30–60 min at room temperature in the dark. Cells were then washed, resuspended in Cell Staining Buffer, and acquired on a BD Celesta flow cytometer (BD Biosciences, San Jose, CA, USA). Data were analyzed using FlowJo software v10 (BD Biosciences). Phosphorylated STAT5 expression was quantified as the median fluorescence intensity (MFI) within the CD3^+^CAR^+^ T-cell population. Results are representative of four independent healthy donor-derived CAR-T cell products (n = 4).

### 4.7. Flow Cytometry

Plant lectin staining was performed using *Sambucus nigra* agglutinin (SNA; Vector Laboratories, Newark, CA, USA) to detect α2,6-linked sialylation. Cells were washed with PBS containing 1% BSA and stained on ice with the respective biotinylated lectins, along with a live/dead marker (Zombie; Biolegend) followed by FITC (Biolegend). CAR detection was performed using a biotin-conjugated goat anti-mouse F(ab′)_2_ antibody (Jackson ImmunoResearch, West Grove, PA, USA) followed by PE-streptavidin (Biolegend). Singlets were gated using FSC-H vs. FSC-A and SSC-H vs. SSC-A, and lymphocytes were identified based on forward/side scatter. Data were reported as the mean fluorescence intensity (MFI ± SEM). Stained cells were immediately acquired using BD FACS Diva 6.1 software and analyzed in FlowJo (BD Biosciences). All experiments were performed 3 times to obtain statistical significance.

### 4.8. ATP Quantification Assay

To assess the effects of galectin-3 on cellular ATP production, Day-10 expanded anti-CD19 CAR-T and enforced ST6GAL1-expressing (ST6) CAR-T cells previously cryopreserved in liquid nitrogen were thawed and cultured in RPMI-1640 supplemented with 10% FBS at a density of 1 × 10^6^ cells/mL. Cells were incubated overnight (16 h) at 37 °C and 5% CO_2_ in the presence or absence of galectin-3 (rhGal-3; R&D Systems) (2 μg/mL ± 50 mM lactose). Following incubation, cells were harvested, washed with PBS, and processed for ATP quantification.

Following incubation, cells were harvested, washed with PBS, and lysed using Tris-EDTA buffer (100 mM Tris, 4 mM EDTA). Cell lysates were boiled for 5 min and subsequently used for ATP quantification. Intracellular ATP levels were measured using the ATP Determination Kit (Invitrogen, A22066) according to the manufacturer’s instructions.

A fresh reaction solution was prepared consisting of dH_2_O, 1× reaction buffer, D-luciferin, recombinant firefly luciferase, and dithiothreitol (DTT). ATP standards ranging from 1 nM to 10 μM were generated in dH_2_O and used to establish a standard curve for ATP quantification. Luminescence assays were performed in flat-bottom 96-well plates with a total reaction volume of 100 μL per well. Blank wells contained 90 μL of reaction solution and 10 μL of dH_2_O. Standard wells contained 90 μL of reaction solution and 10 μL of ATP standard. Sample wells contained 90 μL of reaction solution and 10 μL of cell lysate. Luminescence was measured using a luminescence microplate reader, and ATP concentrations were determined from the corresponding ATP standard curve.

All experimental conditions for each donor were performed in triplicate, and data from independent donor experiments were combined for statistical analysis.

### 4.9. Glucose Uptake Assay

Glucose uptake was measured using the Glucose Uptake-Glo™ Assay (Promega, Madison, WI, USA) according to the manufacturer’s instructions. Day-10 expanded CAR-T and ST6GAL1-overexpressing (ST6OE) CAR-T cells derived from the same healthy donors and previoussly cryopreserved in liquid nitrogen were thawed and cultured overnight (16 h) in RPMI-1640 supplemented with 10% FBS at a density of 1 × 10^6^ cells/mL. Cells were incubated at 37 °C and 5% CO_2_ in the presence or absence of recombinant human galectin-3 (rhGal-3; R&D Systems) (4 μg/mL).

Following incubation, cells were washed with glucose-free PBS to remove residual media, IL-2, and Gal-3 and resuspended in glucose-free PBS. Cells were seeded at 1 × 10^5^ viable cells per well (100 μL) in flat-bottom 96-well plates. Glucose uptake was initiated by the addition of 50 μL of 1 mM 2-deoxy-D-glucose (2DG) prepared in glucose-free PBS and incubation for 10 min at room temperature. Uptake was terminated by the addition of 25 μL of Stop Buffer followed by 25 μL of Neutralization Buffer. Subsequently, 100 μL of freshly prepared 2DG6P Detection Reagent containing luciferase reagent, NADP+, glucose-6-phosphate dehydrogenase (G6PDH), reductase, and reductase substrate was added to each well according to the manufacturer’s protocol. Plates were incubated at room temperature for 0.5 h before luminescence was measured using a luminescence microplate reader.

For each donor and treatment condition, one assay well containing 2DG and three corresponding wells lacking 2DG were included. Wells without 2DG served as background controls. Net glucose uptake was calculated by subtracting the mean luminescence of the no-2DG control wells from the luminescence measured in the corresponding 2DG-treated well. All experimental conditions for each donor were performed in triplicate, and data from independent donor experiments were combined for statistical analysis.

### 4.10. Seahorse Metabolic Activity

To investigate the effect of Gal-3 on metabolic activity, cells were incubated with 2 µg/mL rhGal-3 (R&D systems) with 200 × 10^3^/mL CAR-T cells and ST6 CAR-T cells in complete medium in 96-well plates for 24 h in 200 µL at 37 °C. Cell were assessed using an XF^e^96 Extracellular Flux Analyzer (Agilent Technologies, Santa Clara, CA, USA). Briefly, donor CAR-T and ST6-transfected suspension cells were collected and resuspended at 2 × 10^6^ cells/mL in XF RPMI assay medium (phenol-red free, pH 7.4), supplemented with 10 mM glucose, 1 mM pyruvate, and 2 mM glutamine. Cells were seeded at a density of 100,000 cells per well (50 μL) into an XF^e^96 assay plate pre-coated with poly-D-lysine one day prior. To ensure cell attachment, the plate was centrifuged at 200× *g* for 5 min. After centrifugation, each well was topped up to a final volume of 175 μL with assay medium. The plate was then incubated in a non-CO_2_ incubator at 37 °C for 30 min to equilibrate prior to the assay. For the T-cell Fitness assay (mitochondria stress test), oxygen consumption rates (OCR) were measured following sequential injections of 2.5 μM Oligomycin A, 0.5 μM BAM15, and 0.5 μM Rotenone/Antimycin A. For the glycolytic rate test, extracellular acidification rates (ECAR) were monitored following the addition of 0.5 μM Rotenone/Antimycin A and 50 mM 2-deoxy-D-glucose (2-DG).

### 4.11. Tumoricidal Assay

To evaluate the effects of galectin-3 and JAK2 inhibition on CAR-T cell-mediated cytotoxicity, CAR-T cells and enforced ST6GAL1-expressing CAR-T cells (ST6 CAR-T cells) were co-cultured with human CD19+ Raji-luciferase-expressing (Raji-luc^+^) target cells in 96-well plates (VWR). To investigate the contribution of JAK/STAT signaling to Gal-3-mediated CAR-T cell dysfunction, effector cells were pretreated for 30 min with JAK2 Inhibitor IV (MilliporeSigma/Calbiochem, Cat. No. 420139; 1 μM, Burlington, MA, USA), an aminoindazole compound that selectively inhibits JAK2 kinase activity, before exposure to recombinant human galectin-3 (rhGal-3; 2 μg/mL). Co-cultures were established at an effector-to-target (E) ratio of 20:1 in RPMI-1640 supplemented with 10% heat-inactivated fetal bovine serum (HI-FBS) in a total volume of 100 μL per well.

Following a 16 h incubation at 37 °C in a humidified 5% CO_2_ incubator, tumor cell viability was assessed using Bright-Glo™ Luciferase Assay Reagent (Promega) according to the manufacturer’s instructions. Luminescence was measured using an AMI HT imaging system (Spectral Instruments Imaging, Tucson, AZ, USA) and expressed as relative light units (RLU). Tumor cell killing was calculated relative to tumor-only control wells, with decreased luminescence indicating increased CAR-T cell-mediated cytotoxicity. All experiments were performed using CAR-T cells generated from five independent donors (n = 5), with each condition analyzed in technical triplicate.

### 4.12. Cell Proliferation Assay

Cell proliferation was evaluated using the Cell Counting Kit-8 (CCK-8; Dojindo, Kumamoto, Japan) following the manufacturer’s protocol. Cells were seeded in 96-well plates at a density of 20,000 cells per well and incubated with 2 µg/mL rhGal-3 (R&D systems). Proliferation was assessed at 0, 24, and 48 h by adding 10 µL of CCK-8 reagent to each well and incubating at 37 °C for 3 h. Absorbance was measured at 450 nm using a Cytation 5 microplate reader (BioTek, Winooski, VT, USA). All experiments were performed 3 times to ensure statistical significance.

### 4.13. Gene Expression Analysis of Public Databases

Publicly available transcriptomic datasets were obtained from the Gene Expression Omnibus (GEO) database (https://www.ncbi.nlm.nih.gov/geo/ (accessed on 28 May 2026)), a public repository for high-throughput gene expression data [31]. The dataset GSE223655 was analyzed to compare gene expression profiles from patients with relapsed/refractory diffuse large B-cell lymphoma (DLBCL) treated with CD19/CD20 tandem chimeric antigen receptor (TanCAR)-T cell therapy [32]. Clinical response groups included patients who achieved complete remission (CR, n = 12), partial remission (PR, n = 11), and progressive disease (PD, n = 12). For differential expression and pathway analyses, CR and PD samples were compared to identify transcriptional programs associated with favorable versus poor clinical outcomes. Differential gene expression analysis was performed using GEO2R (https://www.ncbi.nlm.nih.gov/geo/geo2r/ (accessed on 28 May 2026)), which utilizes the limma package in R for statistical analysis [33]. Genes were ranked according to the log2 fold change derived from the GEO2R output, and an adjusted *p*-value < 0.05 was considered statistically significant.

To identify biological pathways associated with the clinical response, gene set enrichment analysis (GSEA) was performed using GSEA Desktop version 4.4 (Broad Institute, Cambridge, MA, USA; https://www.gsea-msigdb.org/gsea/index.jsp (accessed on 28 May 2026)) as previously described [34]. A preranked gene list generated from GEO2R differential expression results was used as input for GSEA. Gene set enrichment was evaluated using the Hallmark gene set collection from the Molecular Signatures Database (MSigDB) [35]. Normalized enrichment scores (NES), nominal *p*-values, false discovery rate (FDR) q-values, and family-wise error rate (FWER) *p*-values were calculated for each gene set. Gene sets with an FDR q-value < 0.25 were considered significantly enriched. Pathways of particular interest, including IL2/STAT5 signaling, inflammatory response, apoptosis, glycolysis, and oxidative phosphorylation, were further evaluated based on enrichment statistics and leading-edge gene analysis. Heatmaps were generated using Morpheus (https://software.broadinstitute.org/morpheus (accessed on 28 May 2026)), and graphical visualizations were created using GraphPad Prism 10 (GraphPad Software, San Diego, CA, USA).

### 4.14. Statistical Analysis

Statistical analyses were performed using GraphPad Prism version 8 (GraphPad Software, San Diego, CA, USA). Data are presented as the mean ± SEM unless otherwise indicated. Normality was assessed using the Shapiro–Wilk test. Comparisons between two groups were performed using unpaired two-tailed Student’s *t*-tests for normally distributed data or Mann–Whitney U tests for non-normally distributed data. SOCS1 Western blot analyses were evaluated using the Wilcoxon matched-pairs signed-rank test. Phospho-STAT5 time-course experiments were analyzed using two-way analysis of variance (ANOVA) followed by Sidak’s multiple comparisons test. Proliferation assays were analyzed using mixed-effects models. Seahorse metabolic assays were analyzed using paired t-tests controlling for donor-to-donor variability. ATP quantification and glucose uptake assays were analyzed using paired Student’s *t*-tests. Tumoricidal assays involving six experimental conditions were analyzed using repeated-measures one-way ANOVA followed by Sidak’s multiple comparisons test. For gene set enrichment analysis (GSEA), statistical significance was determined using normalized enrichment scores (NES), nominal *p*-values, and false discovery rate (FDR) q-values generated using GSEA Desktop version 4.4. Gene sets with an FDR q-value < 0.25 were considered significantly enriched. A *p*-value < 0.05 was considered statistically significant.

## 5. Conclusions

This study identifies STAT5/SOCS signaling and metabolic fitness as important downstream mechanisms associated with the protective effects of ST6GAL1-mediated glycoengineering in CAR-T cells. We demonstrate that Gal-3 induces dysregulated STAT5 activation, suppresses glycolytic and mitochondrial metabolism, and impairs CAR-T cell function. In contrast, enforcing ST6GAL1 expression enhanced SOCS1 and SOCS3 expression, attenuated STAT5 activation, preserved bioenergetic fitness, and improved resistance to Gal-3-mediated suppression. These findings are consistent with the emerging understanding that cytokine signaling and metabolic programming are critical determinants of CAR-T cell persistence and therapeutic efficacy [27,36]. Furthermore, the pharmacologic inhibition of JAK/STAT signaling partially restored CAR-T cell cytotoxicity, supporting a functional role for this pathway in Gal-3-induced dysfunction. An analysis of an independent clinical CAR-T transcriptomic dataset further demonstrated the enrichment of IL-2/STAT5 signaling and inflammatory response pathways in patients achieving complete remission, highlighting the clinical relevance of these findings. Collectively, these results support a model in which α2,6-sialylation protects CAR-T cells from Gal-3-mediated immunosuppression by preserving cytokine signaling homeostasis and metabolic fitness. Given the established role of galectins as regulators of tumor immune evasion [11,37], our findings further establish glycoengineering as a promising strategy to improve CAR-T cell persistence and antitumor efficacy in galectin-rich tumor microenvironments. Future studies investigating the interplay between glycosylation, cytokine signaling, and cellular metabolism may provide new opportunities to engineer next-generation CAR-T cell therapies with improved durability and efficacy against both hematologic and solid malignancies.

## Data Availability

The data analyzed in this study is publicly available through the Genomic Data Commons (GDC) Data Portal (https://portal.gdc.cancer.gov/ (accessed on 28 May 2026)) and the Gene Expression Omnibus (GEO) database at GSE223655.

## Supplementary Materials

The following supporting information can be downloaded at: https://www.mdpi.com/article/10.3390/ijms27146393/s1.

## Abbreviations

The following abbreviations are used in this manuscript:

ATP	Adenosine Triphosphate
CAR	Chimeric Antigen Receptor
CR	Complete Remission
CSF2RB	Colony Stimulating Factor 2 Receptor Beta
DLBCL	Diffuse Large B-Cell Lymphoma
ECAR	Extracellular Acidification Rate
FDA	Food and Drug Administration
FDR	False Discovery Rate
Gal-3	Galectin-3
GEO	Gene Expression Omnibus
GSEA	Gene Set Enrichment Analysis
HI-FBS	Heat-Inactivated Fetal Bovine Serum
IL-2	Interleukin-2
IL5RA	Interleukin-5 Receptor Alpha
JAK	Janus Kinase
MFI	Mean Fluorescence Intensity
MHC	Major Histocompatibility Complex
MOI	Multiplicity of Infection
NES	Normalized Enrichment Score
OCR	Oxygen Consumption Rate
PBMC	Peripheral Blood Mononuclear Cell
PD	Progressive Disease
PR	Partial Remission
qPCR	Quantitative Polymerase Chain Reaction
rhGal-3	Recombinant Human Galectin-3
RT-qPCR	Reverse Transcription Quantitative Polymerase Chain Reaction
scFv	Single-Chain Variable Fragment
SEM	Standard Error of the Mean
SNA	Sambucus nigra Agglutinin
SOCS1	Suppressor of Cytokine Signaling 1
SOCS3	Suppressor of Cytokine Signaling 3
STAT5	Signal Transducer and Activator of Transcription 5
STAT5A	Signal Transducer and Activator of Transcription 5A
STAT5B	Signal Transducer and Activator of Transcription 5B
ST6	ST6GAL1-Overexpressing CAR-T Cells
ST6GAL1	β-Galactoside α2,6-Sialyltransferase 1
TanCAR	Tandem Chimeric Antigen Receptor
TME	Tumor Microenvironment
