# Supplementary material for: ST6GAL1 Glycoengineering Rewires Cytokine Signaling and Preserves Metabolic Fitness in CAR-T Cells Under Galectin-3-Mediated Immunosuppression
Source: Int J Mol Sci. 2026 Jul 18;27(14):6393. doi: 10.3390/ijms27146393 (PMC13409972; doi:10.3390/ijms27146393)
Supplement: Supplementary file 1 [file ijms-27-06393-s001.zip › ijms-4405127-supplementary.pdf]

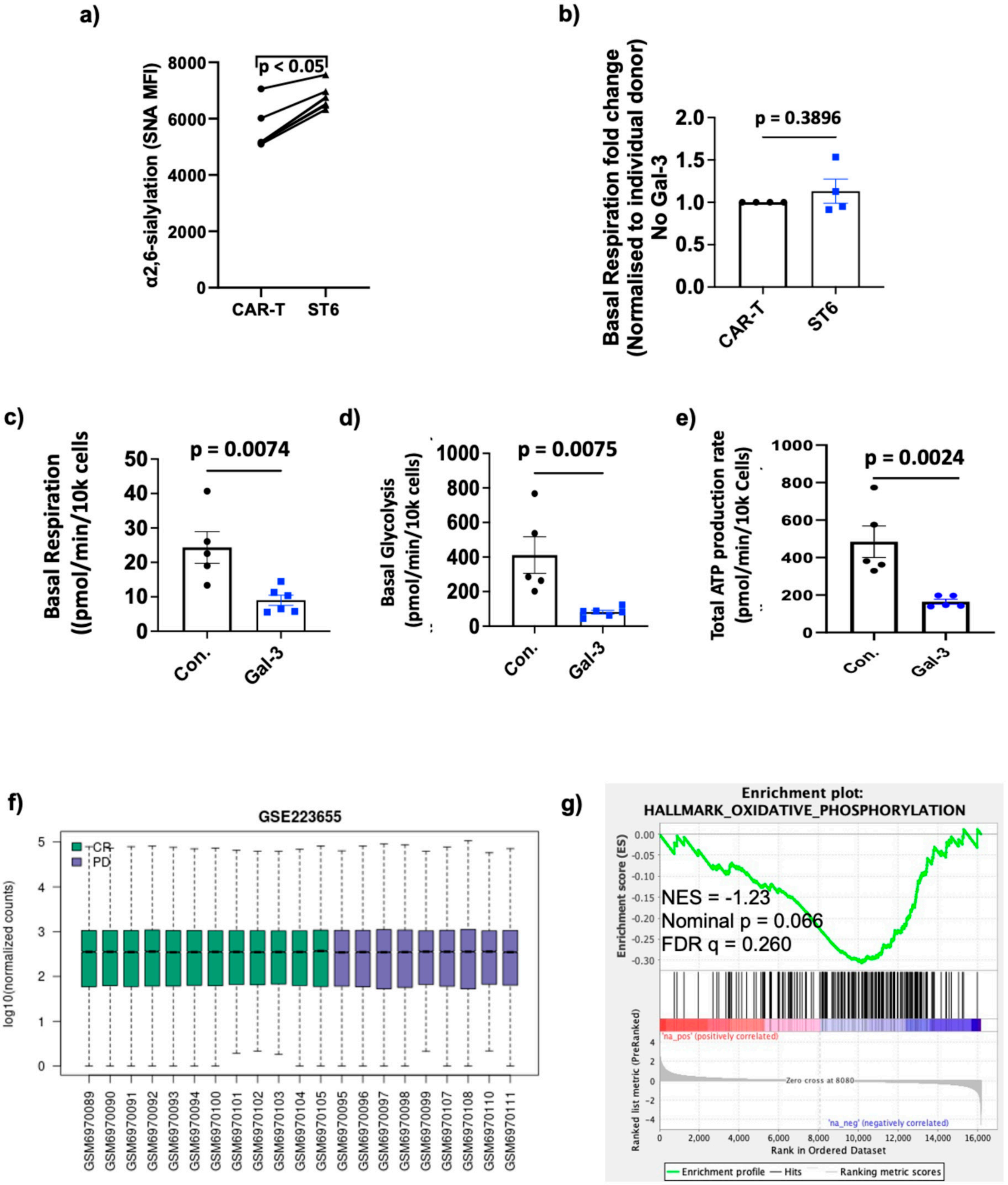

Figure S1. Validation of ST6GAL1 overexpression and transcriptomic quality control analyses.

(a) Flow cytometry confirmed increased SNA-*α*2,6 sialylation binding in ST6GAL1-overexpressing CAR-T cells (ST6 CAR-T) compared to conventional CAR-T cells, validating successful ST6GAL1-mediated glycoengineering ($p < 0.05$, paired t-test). **(b)** Under basal (no Gal-3) conditions, ST6GAL1 overexpression did not significantly alter basal respiration fold change relative to conventional CAR-T cells (p = 0.3896, unpaired t-test), indicating that ST6 CAR-T cells retained normal metabolic activity in the absence of Gal-3. **(c–e)** Treatment with rhGal-3 induced substantial metabolic suppression

in CAR-T cells, with significant reductions in basal mitochondrial respiration ($p = 0.0074$), basal glycolysis ($p = 0.0075$), and total ATP production rate ($p = 0.0024$), all determined by unpaired t-test. **(f)** Boxplot analysis of normalized gene expression distributions from the GSE223655 dataset showed highly similar distributions across complete responder (CR) and progressive disease (PD) samples, confirming successful correction of technical variation and suitability of the dataset for downstream differential expression and gene set enrichment analyses. **(g)** Gene set enrichment analysis (GSEA) revealed a trend toward reduced oxidative phosphorylation in PD samples relative to CR samples; however, this enrichment did not reach statistical significance (NES = -1.23, nominal $p = 0.066$, FDR $q = 0.260$, GSEA permutation test).
